# Somatic Mutations in Surgically Treated Colorectal Liver Metastases: An Overview

**DOI:** 10.3390/cells13080679

**Published:** 2024-04-14

**Authors:** Jane Wang, Julia Botvinov, Aarshvi Jahnvi Bhatt, Katharina Beyer, Martin E. Kreis, Mohamed Adam, Adnan Alseidi, Georgios Antonios Margonis

**Affiliations:** 1 Department of Surgery, University of California San Francisco, San Francisco, CA 94143, USA; mohamed.adam@ucsf.edu (M.A.); adnan.alseidi@ucsf.edu (A.A.); 2 Hackensack Meridian School of Medicine, Nutley, NJ 07110, USA; julia.botvinov@hmhn.org; 3 University of Toledo College of Medicine and Life Sciences, Toledo, OH 43614, USA; aarshvi.bhatt@rockets.utoledo.edu; 4 Department of General and Visceral Surgery, Charité Campus Benjamin Franklin, 12203 Berlin, Germany; katharina.beyer2@charite.de (K.B.); martin.kreis@charite.de (M.E.K.); 5 Department of Surgery, Memorial Sloan Kettering Cancer Center, New York, NY 10065, USA; margonig@mskcc.org

**Keywords:** CRLM, somatic mutations, *RAS*, *BRAF*

## Abstract

Colorectal cancer is the second most common cause of cancer death in the United States, and up to half of patients develop colorectal liver metastases (CRLMs). Notably, somatic genetic mutations, such as mutations in *RAS*, *BRAF*, mismatch repair (MMR) genes, *TP53*, and *SMAD4*, have been shown to play a prognostic role in patients with CRLM. This review summarizes and appraises the current literature regarding the most relevant somatic mutations in surgically treated CRLM by not only reviewing representative studies, but also providing recommendations for areas of future research. In addition, advancements in genetic testing and an increasing emphasis on precision medicine have led to a more nuanced understanding of these mutations; thus, more granular data for each mutation are reviewed when available. Importantly, such knowledge can pave the way for precision medicine with the ultimate goal of improving patient outcomes.

## 1. Introduction

Colorectal cancer is the second most common cause of cancer death in the United States, with an estimated 52,550 deaths in 2023 alone [1]. Notably, up to half of these patients develop colorectal liver metastasis (CRLM), which remains the primary cause of death for those with colorectal cancer [2,3]. Surgical resection offers the only chance for a cure; however, long-term survival remains modest at best, with a prior systematic review and meta-analysis showing a median overall survival of 43.2 months after resection of the CRLM [4]. This modest survival rate may be explained by the frequent and early recurrences following CRLM resection, which are not always amenable to a repeat hepatectomy [5]. In fact, one series noted a median recurrence-free survival of only 16 months [6]. On the other hand, some patients with resected CRLM may be cured of disease, with one study reporting a cure rate of 20% at ten years after surgery [7]. This variability in prognosis may be explained by the intrinsic heterogeneity of cancer [8]. Thus, the ability to stratify patients based on their individual characteristics is necessary to provide personalized treatments.

As precision medicine has gained traction in the medical field, previous studies have aimed to identify prognostic factors for CRLM patients [9,10,11]. Among these, somatic genetic mutations, such as mutations in *RAS*, *BRAF*, mismatch repair (MMR) genes, *TP53*, and *SMAD4*, are of particular interest, as understanding a patient’s unique genetic profile can pave the way for individualized treatment. Although the importance of these somatic mutations is well established, there has been a recent surge in publications on this topic that describe several novel findings. Specifically, more recent studies have shown that the prognostic role of these somatic mutations is not monolithic in nature; rather, it depends on numerous other clinical factors, such as the primary tumor location, the specific nucleotide affected, and the presence of co-mutations. Such advances in knowledge may be key to optimizing patient care and advancing personalized medicine. Thus, an updated, contemporary review of the literature is warranted.

In this context, our aim is to appraise the current literature on somatic mutations in surgically treated CRLM by not only providing an overview of representative studies in the field, but also reviewing novel findings. Specifically, we focused on the literature surrounding *RAS*, *BRAF*, MMR genes, *TP53*, and *SMAD4*. For *RAS* and *BRAF*, we identified the largest and/or the most recently published studies for each sub-topic. For the other somatic mutations, we provided a more general overview of existing data given that the literature is more limited. For topics with conflicting viewpoints in the literature, we discussed relevant meta-analyses if available and also emphasized areas where further research is needed. This review falls under the “overview” review type [12]. 

## 2. *RAS* in CRLM

**Figure 1:**
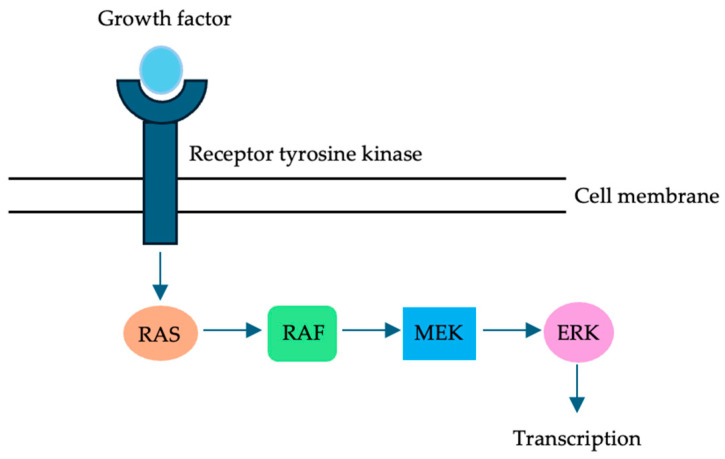
The RAS family of proteins are GTPases that help regulate cellular proliferation, differentiation, and survival. BRAF, which is part of the same signaling pathway as RAS, is a serine/threonine kinase that plays an essential role in the MAPK signaling pathway. It helps regulate cell growth and survival.

The RAS family of proteins, encoded by the *KRAS*, *NRAS*, and *HRAS* genes, are GTPases that help regulate cellular proliferation, differentiation, and survival (Figure 1) [13]. *KRAS* mutations, typically seen on codons 12, 13, or 61, result in constitutive activation of the KRAS protein, which can ultimately lead to cancer [14]. *KRAS* mutations are common in CRLM, with the reported prevalence ranging from 25% to 52% [15]. 

### 2.1. The Prognostic Role of RAS Mutations

Many studies present the *RAS* mutation as a binary variable (i.e., *RAS*-mutated CRLM vs. *RAS* wild-type CRLM). The largest series to date used the National Cancer Database to identify 1116 patients with *KRAS*-mutated CRLM who underwent resection of both their primary tumor and metastatic disease. Compared to their wild-type counterparts, patients with *KRAS* mutations had significantly lower five-year overall survival (31% vs. 42%; *p* < 0.001). Furthermore, the presence of a *KRAS* mutation was found to be independently associated with worse overall survival (hazard ratio [HR] 1.21, *p* = 0.012) [16]. While most existing studies have similarly reported a negative prognostic role of *RAS* mutations in overall survival, a few studies reported no such effect [15,17]. Notably, a meta-analysis published in 2021 confirmed a negative prognostic impact of *KRAS* mutations on overall survival and recurrence-free survival (HR 1.5 and 1.36, respectively) [18].

### 2.2. The Prognostic Role of RAS Mutations on a Granular Level

Some studies have investigated the prognostic role of *KRAS* mutations on a more granular level. In the largest study on this topic to date, Olthof et al. investigated *KRAS* mutations on the exon-, codon-, and nucleotide-specific levels in an international cohort of patients with CRLM [19]. The authors divided the various *KRAS* mutations into high- vs. low-risk groups as defined by their overall survival rates relative to those of the entire cohort. They found that patients with high-risk *KRAS* mutations, such as G12A and G12V, had lower median overall survival than those with low-risk *KRAS* mutations, such as G12C and G12D (34.2 months vs. 53.1 months, respectively, *p* < 0.001). On multivariable analysis, high-risk *KRAS* mutations remained associated with worse overall survival compared to low-risk mutations (HR 1.44, *p* < 0.003). Interestingly, the authors found no significant difference in patient outcomes on the codon-specific level (codon 12 vs. 13). This is consistent with a prior study from the MD Anderson Cancer Center [20] but contradicts the results of a prior study from the Johns Hopkins Hospital [21]. No meta-analyses have yet been conducted on this topic. 

### 2.3. RAS Mutations Do Not Function in a Vacuum: Context Matters

Other disease factors, such as primary tumor laterality, receipt of pre-hepatectomy chemotherapy, and timing of metastatic disease, may impact the prognostic role of *KRAS* mutations. Regarding primary tumor laterality, previous studies have shown that right-sided tumors are associated with worse overall survival than left-sided tumors [22,23]. Subsequent studies explored this relationship in the context of mutational status. For example, Belias et al. conducted a systematic review and meta-analysis that included 7475 patients across eight studies and investigated whether the prognostic role of primary tumor laterality is dependent on *KRAS* mutational status. Interestingly, they found that right-sided tumors did indeed confer a worse survival for patients with CRLM but only in those with wild-type disease (left-sided disease, pooled HR 0.71) [24]. 

Regarding pre-hepatectomy chemotherapy, a study from the Johns Hopkins Hospital reported that the *KRAS* mutation was prognostic only among patients who received preoperative chemotherapy (HR 1.67, *p* = 0.012) [25]. One possible explanation relates to the findings of another study, which noted that the receipt of adjuvant FOLFOX for primary colorectal cancer may exert a selective pressure favoring a chemotherapy-resistant subset of disease enriched for *KRAS* mutations, while preventing liver recurrences for patients with *KRAS* wild-type primary tumors [26]. It is unknown whether the frequency of the more “aggressive” *KRAS* point mutations changes following the receipt of pre-hepatectomy chemotherapy. 

Finally, in regard to the timing of metastatic disease, Sakai et al. found that in a cohort of 101 patients, *KRAS* mutation was independently associated with worse overall survival for patients with synchronous CRLM but not in those with metachronous disease (synchronous disease, HR 4.316, *p* < 0.001) [27]. No meta-analyses have yet been published on the relationship between *KRAS* mutations and chemotherapy or timing of metastatic disease.

### 2.4. RAS Mutations Can Inform Treatment: Optimal Surgical Margin Width

*RAS* mutations have also been found to impact the optimal surgical resection margin. For example, Brudvik et al. concluded that *RAS* mutation is significantly associated with positive surgical margins (R1) (HR 2.44, *p* = 0.005) and recommended pursuing a 1 cm margin if possible [28]. In contrast, Margonis et al. found that no amount of margin clearance (including a margin width of ≥1 cm) was associated with improved survival compared to an R1 resection in the presence of a *KRAS* mutation. As the authors noted, this likely reflects the poor disease biology conferred by the *KRAS* mutation which extends beyond tumor behavior on a local level [29]. Most recently, Bertsimas et al. utilized a novel, artificial intelligence-based approach called optimal policy trees and determined the optimal margin width in *KRAS*-mutated CRLM to be 7 mm [30]. Notably, the area under the curve of their prediction model was 0.76, which is the highest ever reported. No meta-analyses have been conducted on this topic, and there is still no consensus on the optimal margin width.

### 2.5. RAS Mutations Can Inform Treatment: Optimal Surgical Technique

Margonis et al. also examined whether the choice of surgical technique, specifically anatomic vs. nonanatomic liver resection, impacts survival in patients with *KRAS*-mutated CRLM. The authors found that nonanatomic resections were associated with worse disease-free survival but only in those with *KRAS*-mutated CRLM, once again highlighting the more aggressive nature of *KRAS*-mutated disease [31]. In contrast, a study conducted by Joechle et al. questioned the benefit of anatomic resections in *KRAS*-mutated CRLM. They concluded that both nonanatomic and anatomic resections provide similar outcomes regardless of *KRAS* mutation status [32]. However, a recently published meta-analysis by Pikoulis et al. noted that anatomic resections do indeed benefit patients with *KRAS*-mutated CRLM, as it was linked to a 40% increase in liver-specific disease-free survival [33].

### 2.6. RAS Mutations Can Inform Treatment: Response to Chemotherapy

Beyond surgical management, *KRAS* mutational status has also been shown to impact the response to neoadjuvant chemotherapy for patients undergoing hepatectomy for CRLM. Zimmitti et al. showed that patients with *KRAS*-mutated CRLM had a lower likelihood of achieving a major pathologic response or an optimal morphologic response compared to their wild-type counterparts [34]. No meta-analyses have been conducted on this topic.

### 2.7. RAS Mutations Can Inform Post-Hepatectomy Surveillance

Finally, *RAS* mutations have also been shown to impact patterns of recurrence after resection of CRLM. Kemeny et al. found that patients who underwent resection of *KRAS*-mutated CRLM had significantly higher rates of bone, brain, and lung metastases than those with wild-type CRLM; interestingly, the rate of liver recurrence was not significantly different between the two groups [35]. Importantly, understanding such patterns could help inform disease surveillance. For example, Kawaguchi et al. determined that patients with *RAS*-mutated CRLM had an intermediate to high risk of liver and lung recurrence for the first four years after surgery. Thus, they recommended intensive surveillance for these patients during this time frame [36]. No meta-analyses have been conducted on this topic.

Ultimately, *RAS* mutations are among the best studied somatic mutations in CRLM and have been shown to have a significant prognostic role on multiple levels. It is imperative that future studies account for *RAS* mutational status given its clear role in outcomes.

## 3. *BRAF* in CRLM

V-RAF murine sarcoma viral oncogene homolog B1 (BRAF), which is part of the same signaling pathway as KRAS, is a serine/threonine kinase that plays an essential role in the mitogen-activated protein kinase (MAPK) signaling pathway and helps regulate cell growth and survival (Figure 1) [37]. Despite the fact that *BRAF* mutations are much rarer than their *RAS* counterparts, they have also been shown to be a poor prognostic indicator. For example, although *BRAF* mutations have been identified in up to 15% of all patients with colorectal cancer, their prevalence among patients undergoing resection of their CRLM is much lower at 0.6% to 5.5% [38]. This likely reflects the fact that most patients with *BRAF*-mutated CRLM present with unresectable disease, alluding to a particularly aggressive disease biology. 

### 3.1. The Prognostic Role of BRAF

Similar to *KRAS*, many studies present the *BRAF* mutation as a binary variable (i.e., *BRAF*-mutated CRLM vs. *BRAF* wild-type CRLM). Importantly, patients with *BRAF*-mutated CRLM may have worse outcomes than both patients with wild-type and *KRAS*-mutated disease. For example, Schirripa et al. identified 309 patients who underwent curative intent resection of their CRLM, of whom 12 had *BRAF* mutations (*RAS* mutation, *n* = 160; wild-type, *n* = 137). On multivariable analysis, they found that patients with *BRAF* mutations had a higher risk of recurrence than patients with *KRAS* mutations or wild-type disease (HR 2.06 and 2.31, respectively, *p* < 0.05). A similar trend was seen for overall survival, although it was no longer significant on multivariable analysis [17]. 

As highlighted by this study, a notable challenge in conducting research on this topic is the rarity of the *BRAF* mutation. However, since then, additional studies with larger cohorts have been published. Bachet et al. published the largest series to date comparing patients with surgically treated, *BRAF*-mutated CRLM to those with resected wild-type disease (*n* = 66 and 183, respectively). On multivariable analysis, the authors found that *BRAF* mutations were associated with worse overall survival (HR 2.21, *p* = 0.041) but not disease-free survival (HR 1.16, *p* = 0.547) [39]. In contrast, Margonis et al. found that *BRAF* mutations were associated with worse disease-free survival on multivariable analysis (HR 2.04, *p* = 0.002), although this was only true for patients with V600E *BRAF*-mutated CRLM [40]. Notably, this debate was resolved by a meta-analysis published in 2021, which identified 232 patients across 13 studies who underwent hepatectomy for *BRAF*-mutated CRLM. *BRAF* mutations were found to be associated with worse overall and disease-free survival (odds ratio [OR] 1.981 and 1.49, respectively) than patients with wild-type disease [38]. 

### 3.2. The Prognostic Role of BRAF on a Granular Level

Recent studies have investigated the prognostic role of *BRAF* mutations from a more granular perspective. For example, in the largest study on this topic to date, Margonis et al. investigated the impact of V600E vs. non-V600E *BRAF* mutations on survival [41]. They found that patients with V600E mutations had a shorter median overall survival than those with non-V600E mutations (30.6 vs. 144 months, respectively, *p* = 0.002); importantly, the presence of a V600E mutation remained independently associated with a higher risk of death on multivariable analysis (HR 3.5, *p* = 0.002). Interestingly, the median overall survival of the patients with non-V600E mutations (144 months) exceeded the median overall survival previously reported for patients with wild-type *BRAF* (60–81 months). This highlights the importance of accounting for codon-specific data when investigating the prognostic role of *BRAF* mutations. Of note, an earlier study by Gagniere et al. concluded that V600E mutations are not associated with worse overall or recurrence-free survival [42]. Despite these conflicting data, there is currently no meta-analysis that explores this topic.

### 3.3. BRAF vs. KRAS/BRAF Co-Mutation

Previously thought to be mutually exclusive, *KRAS*/*BRAF* co-mutations have been described in the literature. In the largest study to date, Margonis et al. performed a survival analysis on a cohort of 17 patients with *KRAS*/*BRAF* co-mutations; interestingly, there was no significant difference in overall or recurrence-free survival between these patients and those with *BRAF* mutations alone [41]. Thus, the presence of both mutations does not appear to have a synergistic, deleterious effect on long-term outcomes. Understandably, no meta-analyses have yet been conducted on this topic given the rarity of this co-mutation.

### 3.4. Is BRAF a “Biologic” Contraindication to Surgery?

A 2021 study from Japan found that survival outcomes of patients with technically resectable, V600E *BRAF*-mutated CRLM were as poor as those with unresectable disease. Thus, the authors concluded that the mere presence of a V600E *BRAF* mutation should be an absolute contraindication to surgery for patients with technically resectable CRLM [43]. However, the authors had only five patients with *BRAF*-mutated CRLM in their cohort, and they did not control for confounding variables. This is a significant limitation since patients who undergo surgery typically have more favorable characteristics than their medically treated counterparts. Notably, a recent multi-institutional study, which is the largest study on this topic to date, arrived at different conclusions. Specifically, Margonis et al. found that surgical treatment is associated with significantly better outcomes than systemic therapy alone for patients with V600E *BRAF*-mutated CRLM. Importantly, the authors addressed confounding by performing propensity score matching of 51 surgically and 51 medically treated patients based on tumor burden and disease characteristics. No meta-analyses have been conducted on this topic.

### 3.5. BRAF and Disease Recurrence

The impact of *BRAF* mutations on postoperative recurrence has also been studied. While a prior study concluded that *BRAF* mutations do not increase the risk of recurrence [39], a subsequent meta-analysis found that the risk of both intra- and extrahepatic recurrence was significantly higher in patients with *BRAF* mutations [38]. Importantly, patients with liver-limited recurrence may benefit from repeat hepatectomy even in the presence of a *BRAF* mutation. Specifically, these patients have been shown to have a longer median overall survival compared to those with liver-limited recurrence who do not undergo surgery (41 vs. 18.7 months, *p* = 0.004) [41]. In summary, *BRAF* mutations are relatively rare in patients who present with resectable CRLM and have been shown to play a negative prognostic role. 

## 4. MMR Genes in CRLM

**Figure 2:**
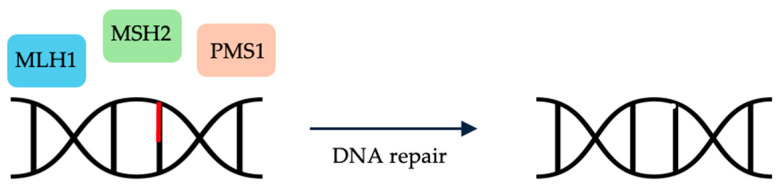
Mismatch repair genes, such as *MLH1*, *MSH2*, and *PMS1*, correct nucleotide base mispairings.

MMR genes, such as *MLH1*, *MLH3*, *MSH2*, *MSH3*, *MSH6*, *PMS1*, and *PMS2*, correct nucleotide base mispairings [44] (Figure 2). Cells that are deficient in these MMR genes accumulate DNA abnormalities throughout the genome; short sequences of repeating nucleotide bases, otherwise known as microsatellites, are also affected [45]. Tumors with a high number of mutations within these microsatellites are deemed microsatellite instable (MSI) cancers, while tumors without these mutations are considered microsatellite stable (MSS). 

A prior systematic review noted a significantly better prognosis for colorectal cancer patients with MSI tumors compared to those with MSS tumors [46]. However, the literature on the prognostic value of MSI status in CRLM is more limited. In the largest and most recent study to date, Turner et al. published a series on 427 patients with MSI CRLM who underwent hepatectomy (along with 2316 patients with MSS tumors) [47]. The authors found that patients in the MSI group had worse overall survival compared to those in the MSS group (33 vs. 41 months, *p* < 0.01). This held true on multivariable analysis, as MSI status was found to be independently associated with worse overall survival (HR 1.21, *p* = 0.04). The authors note that one possible explanation could be early disease recurrence after hepatectomy for patients with MSI-high tumors. This phenomenon has been previously described by Matteo et al. [48]. No meta-analyses have been conducted on this topic. 

## 5. *TP53* and *SMAD4* in CRLM

**Figure 3:**
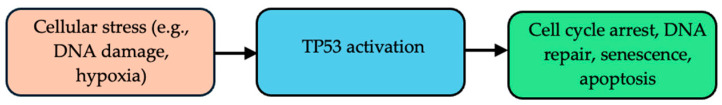
*TP53* is a tumor suppressor gene that plays a key role in the cell cycle. In the presence of cellular stress, it promotes cell cycle arrest to allow for cellular apoptosis vs. DNA repair.

**Figure 4:**
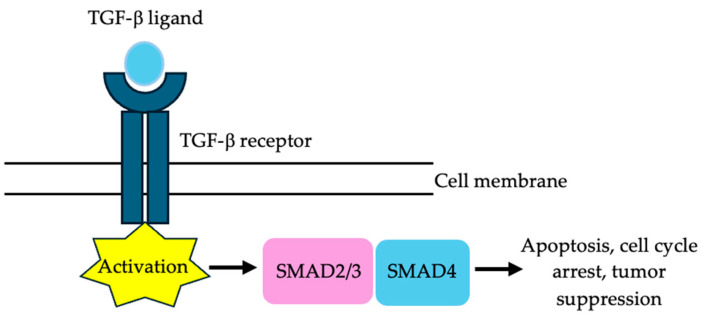
*SMAD4* is a tumor suppressor gene that mediates the transforming growth factor beta (TGF-β) signaling pathway. It can induce cell cycle arrest and apoptosis.

Tumor protein p53 (*TP53*), which is the most frequently mutated gene in human cancers, is a tumor suppressor gene that has a prevalence of 36% to 65.6% and plays a key role in the cell cycle (Figure 3). Mothers against decapentaplegic homolog 4 (*SMAD4*), which is also a tumor suppressor gene, has a prevalence of approximately 15% and induces cell cycle arrest and apoptosis (Figure 4) [15,49]. 

### 5.1. The Prognostic Role of TP53

The literature regarding the prognostic role of *TP53* in CRLM is conflicting. Chun et al. identified a cohort of 401 patients who underwent resection of their CRLM, of whom 65.6% had *TP53* mutations. They found that *TP53* mutations in isolation were not significantly associated with overall survival [50]. Frankel et al. reached a similar conclusion [51]. In contrast, Cha et al. concluded that *TP53* mutations were independently associated with worse overall survival (HR 1.27, *p* = 0.033), although only in CRLM of rectal origin [52]. Mollevi et al. similarly concluded that *TP53* mutational status was a poor prognostic indicator [53]. A study on patients with colorectal cancer may provide some insight into these conflicting results. Specifically, the authors determined that tumor site, mutation type, and adjuvant treatment may impact the exact prognostic role of *TP53* [54]. Thus, although this study was not conducted in patients with CRLM, it is very possible that similar relationships exist in this patient population; the findings by Cha et al. already support this hypothesis. Thus, a more granular investigation of *TP53* is needed. 

Of note, although Chun et al. noted that *TP53* mutations were not significantly associated with overall survival, they did find that patients with *RAS*/*TP53* co-mutations had worse five-year overall survival compared to those with wild-type *TP53* (12.2% vs. 55.7%, respectively, *p* < 0.001) [50]. Lillemoe et al. also found that *RAS*/*TP53* co-mutations were independently associated with worse overall survival compared to those without the co-mutation (HR 2.8, *p* = 0.04) [55]. Similarly, Cha et al. found on multivariable analysis that a *RAS*/*TP53* co-mutation was associated with the worst outcomes as compared to *RAS* and *TP53* mutations in isolation (HR 1.81; *p* < 0.001) [52]. This alludes to the presence of complex relationships among somatic mutations and emphasizes the importance of investigating co-mutations when possible. No meta-analyses have been conducted on this topic.

### 5.2. The Prognostic Role of SMAD4

*SMAD4* mutations have generally been found to have a negative prognostic role in patients with CRLM. Lopez-Gomez et al. concluded that the *SMAD4* mutation was independently associated with risk of recurrence (HR 1.68, *p* = 0.032), although it was not found to be associated with overall survival even on univariable analysis [56]. In a more recent study, Mizuno et al. identified a cohort of 278 patients who underwent resection of their CRLM, of whom 37 had *SMAD4* mutations. Compared to patients with wild-type disease, those with *SMAD4*-mutated CRLM had worse three-year overall survival (62% vs. 82%, *p* < 0.0001). On multivariable analysis, the *SMAD4* mutation was found to be an independent predictor of poor overall survival (HR 2.77, *p* < 0.0001). In addition, although there was no association between *SMAD4* mutational status and recurrence rates, the authors did find that patients with *SMAD4* mutations had significantly lower rates of repeat hepatectomy compared to those with wild-type disease (25% vs. 47%, respectively, *p* = 0.042) [57]. One possible explanation is that *SMAD4* mutations confer a more aggressive tumor biology and thus recurrence presents more frequently as unresectable disease. It is unclear why the results of these two studies are contradictory (i.e., Lopez-Gomez et al. concluded that *SMAD4* impacts recurrence but not overall survival, whereas Mizuno et al. reported that *SMAD4* affects overall survival but not recurrence). However, it is worth noting that the former had a smaller overall sample size (*n* = 121), and the authors did not specify what proportion of these patients had *SMAD4* mutations. No meta-analyses have been conducted on this topic, although one has been performed for colorectal cancer [58].

### 5.3. The Impact of TP53 and SMAD4 Co-Mutations

Interestingly, *TP53* and *SMAD4* mutations may impact the prognostic role of other somatic mutations. For example, Kawaguchi et al. found that while a *TP53*/*SMAD4*/*RAS* triple mutation was linked with worse overall and recurrence-free survival, patients with a *RAS* mutation combined with wild-type *TP53*/*SMAD4* had similar overall and recurrence-free survival as those with *TP53*/*SMAD4*/*RAS* wild-type disease. This suggests that examining *RAS* mutations in isolation may not be sufficient to accurately gauge prognosis [59]. In another study, Datta et al. found that patients with resected CRLM who had *TP53* and *RAS*/*BRAF* (either *KRAS*, *NRAS*, or *BRAF*) co-mutations had significantly worse overall survival compared to those with single mutations (median overall survival; co-mutation: 40 months; *TP53* only: 132 months; *RAS*/*BRAF* only: 65 months, *p* < 0.0001) [60]. This suggests that there may be a synergistic effect among these mutations. The importance of these rarer somatic mutations and co-mutations was recognized by Lang et al., who refined the modified clinical risk score. Specifically, based on their findings from next-generation sequencing, the authors replaced *RAS* with *RAS*–*RAF* pathway mutational status and added *SMAD* mutational status to the scoring system [61]. No meta-analyses have been conducted on this topic.

## 6. Other Somatic Mutations in CRLM

The prognostic role of other somatic mutations in CRLM, such as adenomatous polyposis coli (*APC*), phosphatidylinositol-4,5-bisphosphate 3-kinase catalytic subunit alpha (*PIK3CA*), phosphatase and tensin homolog (*PTEN*), and caudal-type homeobox 2 (*CDX2*), has been described. For example, Yamashita et al. identified 396 patients who underwent hepatectomy for CRLM, of whom 45 patients had an *APC/PIK3CA* co-mutation. The absence of this double mutation was an independent predictor of major pathologic response to chemotherapy (OR 2.91, *p* = 0.002). In addition, the presence of the *APC/PIK3CA* co-mutation was an independent predictor of worse overall survival compared to those with wild-type *APC* and/or wild-type *PIK3CA* (HR 3.09, *p* < 0.001). Importantly, the prognostic impact of this co-mutation was found to be independent of *RAS* mutational status, as it resulted in worse survival after metastasectomy in both patients with *RAS*-mutated and *RAS* wild-type CRLM [62]. Regarding *PTEN*, Sawai et al. identified a cohort of 69 patients with resected CRLM and found that the five-year survival rate of patients with loss of *PTEN* expression was significantly lower than those who had positive *PTEN* expression (12.7% vs. 64.4%, respectively, *p* < 0.05) [63]. Finally, regarding *CDX2*, Shigematsu et al. published a study investigating the outcomes of 396 patients who underwent hepatectomy for CRLM, of whom 36 (9.1%) had reduced *CDX2* expression [64]. The authors found that patients with reduced *CDX2* expression had worse overall survival than those with high expression (HR 2.41, *p* < 0.001). No meta-analyses have been conducted on this topic. While it is beyond the scope of this review to delve into extensive detail regarding all identified somatic mutations in CRLM, it is important to highlight that this field is continuously evolving.

## 7. Discussion

Somatic mutations play an important role in understanding both outcomes and disease behavior for patients with surgically treated CRLM. Both *RAS* and *BRAF* mutations have largely been shown to have negative prognostic roles, although newer studies investigating these mutations on a more granular level show that such associations may be more nuanced than previously thought [18,19,38,41]. Furthermore, as data regarding mutations such as *TP53* and *SMAD4* are published, it is becoming increasingly apparent that genetic data should not be considered in isolation, as their prognostic impact may be dependent on the presence of other mutations and clinicopathologic factors [59,60]. Finally, data on other mutations such as *PTEN* and *CDX2* are more limited, and their role in CRLM warrants further research [62,63,64]. 

Of course, the question arises as to how studying these somatic mutations can impact clinical practice. The ability to accurately ascertain patient prognosis can not only facilitate the informed consent process, but it can also help clinicians identify the subset of patients with particularly poor prognosis that may not benefit from surgery. Prior groups have developed clinical risk scores that predict various outcomes. For example, the Fong score predicts the risk of recurrence after hepatectomy for CRLM and includes clinicopathologic data such as the primary tumor nodal status, disease-free interval, number and size of CRLM, and carcinoembryonic antigen level [10]. More recently developed risk scores have incorporated genetic data. For example, Brudvik et al. created a modified clinical risk score to predict survival after resection of CRLM and included the primary tumor nodal status, size of the largest CRLM, and *RAS* mutational status [65]. Unfortunately, its predictive ability was rather limited, with a c-index of 0.69. One possible explanation is that current risk scores are unable to capture nonlinear relationships that may exist among predictors. Another potential explanation is that there likely exist unidentified variables of interest and/or confounding factors. As seen by the impact of co-mutations on the prognostic role of *TP53*, it is possible that capturing other somatic mutations may augment a clinical risk score’s ability to predict outcomes. Once optimized, such tools can be used to provide individualized predictions. 

Understanding the prognostic role of somatic mutations in CRLM may also facilitate surgical decision making. As previously discussed, the presence of a *KRAS* mutation may impact the optimal surgical margin width, the ideal surgical technique (e.g., nonanatomic vs. anatomic liver resection), and even the optimal surveillance strategy in the postoperative setting [28,29,30,33,36]. Although similar studies for *BRAF* mutations are lacking due to their relative rarity, research shows that surgery for patients with V600E *BRAF*-mutated CRLM may be reasonable as long as there is no concurrent extrahepatic disease. This is in stark contrast to prior research suggesting that V600E *BRAF* mutations are a biologic contraindication to surgery for patients with otherwise resectable disease [43]. Finally, drug development may certainly benefit from a deeper understanding of somatic mutations; for example, agents targeting *KRAS* mutations are an area of active research [66,67,68,69,70,71,72,73,74].

## 8. Recommendations for Future Research

Regarding areas of future research, studies have already begun to unveil the heterogeneity in both *KRAS* and *BRAF* mutations; specifically, exon-, codon-, and nucleotide-specific mutations have been shown to have distinct prognostic implications. Thus, as genetic data become more widely available, it is imperative that researchers further explore these nuanced relationships. In addition, the relationship among the various somatic mutations warrants additional investigation, as some mutations may not be prognostic in isolation. Furthermore, as alluded to by the limited performance of current clinical risk scores, the relationships between the various predictors are likely multidimensional and too complex to capture with traditional statistical approaches. Thus, methods such as machine learning, which are better equipped to handle nonlinear relationships, should be considered. Finally, there are several areas in which current studies have yielded conflicting results and thus meta-analyses would be of benefit. Table 1 and Table 2 summarize the topics with and without meta-analyses, respectively. 

## 9. Conclusions

In conclusion, various somatic mutations have been identified and described in patients with surgically treated CRLM, and studies have shown that these mutations play a prognostic role on multiple levels. Importantly, understanding these relationships can enhance clinical practice by informing individualized recommendations. Areas of future research include further exploring the more nuanced relationships among the various somatic mutations and utilizing more complex statistical approaches such as machine learning to optimize clinical risk scores. In addition, most studies in CRLM have focused on *RAS* and *BRAF*, so additional studies on other mutations (e.g., *TP53*, *SMAD4*, *APC*, etc.) are warranted. Importantly, given the rarity of some mutations and the fact that multiple sub-analyses are needed to explore these mutations on a more granular level, multi-institutional collaborations will be essential.

## Figures and Tables

**Table 1:** A summary of topics with published meta-analyses.

***KRAS***
*KRAS* mutations have a negative prognostic impact on overall survival and recurrence-free survival [18]. Patients with *KRAS*-mutated CRLM benefit from anatomic resections [33]. The prognostic role of primary tumor laterality is dependent on *KRAS* mutational status [24].
***BRAF***
*BRAF* mutations have a negative prognostic impact on overall survival and recurrence-free survival [38,41,42]. The risk of both intrahepatic and extrahepatic recurrence is significantly higher in patients with *BRAF* mutations [38].

**Table 2:** A summary of topics with no meta-analyses to date.

***KRAS***
What is the prognostic role of *RAS* mutations on an exon-, codon-, and nucleotide-specific level?
What is the optimal surgical margin width for CRLM in the context of *RAS* mutations? How can *RAS* mutations be used to inform post-hepatectomy surveillance? What is the relationship between *KRAS* mutations and chemotherapy, including the response to neoadjuvant chemotherapy? What is the relationship between *KRAS* mutations and the timing of metastatic disease?
***BRAF***
What are the outcomes of patients with *BRAF*-mutated CRLM stratified by V600E and non-V600E mutations?
Should *BRAF* mutations be considered a “biologic” contraindication to surgery? What is the prognostic impact of *KRAS/BRAF* co-mutations?
**Other Mutations**
What is the prognostic role of MMR genes in CRLM? What is the prognostic role of *TP53*, *SMAD4*, and *TP53/SMAD4* co-mutations? What is the prognostic role of other somatic mutations, such as *APC*, *PTEN*, and *CDX2*?
